# Legislation for the Prevention of Blindness

**Published:** 1894-10

**Authors:** 


### LEGISLATION FOR THE PREVENTION OF BLINDNESS.

The next legislature will probably pass the bill for three boards of medical examiners. There is another medical matter which it should consider, in the interests of humanity. It is the enactment of a law for the prevention of blindness.

In 1887 Dr. Lucien Howe, of Buffalo, N. Y., presented before the meeting of the American Ophthalmological Society a short article in regard to the increase of blindness in the United States, calling attention to the fact that according to the census reports the number of blind in this country was increasing much more rapidly than the population. He also noted that a large proportion of this blindness was due to ophthalmia neonatorum, and hence such laws as would decrease the number of cases of ophthalmia in the new-born would decrease the amount of blindness. That such is the lamentable fact is shown by a report recently made by Dr. Osborne in the April number of *Archives of Ophthalmology*, in which he discusses the cause of blindness in Ontario. In an examination made in a blind asylum of thirty-two inmates he found that ophthalmia neonatorum was the cause in sixteen per cent., the highest of all causes except cataract. It was due, however, to Dr. Howe the priority of first calling attention to these facts, and to cause active steps to be taken in different States, looking to the enactment of laws bearing upon this subject. Since that time the following States have taken the matter in hand, and have also passed a law for the prevention of blindness, viz.: New York, Rhode Island, Minnesota, and Maryland. The last State to enact such a law was Ohio, and we had the pleasure of receiving from Dr. Culberson of that State a copy of the law recently passed, and which, in many respects, is the same as that already in other States. For the benefit of those who are not familiar with the purport of this law we reproduce the same:

A Bill for the Prevention of Blindness in the State of Ohio.

SECTION 1. Be it enacted by the General Assembly of the State of Ohio, That should one or both eyes of an infant become inflamed or swollen, or show any unnatural discharge, at any time within ten (10) days after birth, it shall be the duty of the midwife, nurse, or relative having charge of such an infant to report, in writing, within six (6) hours, to the physician in attendance upon the family, or in the absence of an attending physician, to the health officer of the city, village, or township within which the infant is living at the time, or in case there is no such officer, to some practitioner of medicine legally qualified to practice in the State of Ohio, the fact that such inflammation, swelling, or unnatural discharge exists.

SECTION 2. Any failure to comply with the provisions of this Act shall be punished by a fine of not less than ten ($10.00) dollars, nor more than one hundred ($100.00) dollars, or imprisonment for not less than thirty (30) days nor more than six (6) months, or both fine and imprisonment.

SECTION 3. This Act shall take effect and be in force from and after its passage.

Dr. Boerne Bettman, of Chicago, read before the Illinois State Medical Society, in May of this year, a paper urging the passage of such legislation in that State.

A similar paper was read before the South Carolina Medical Society in April last, by Dr. Charles W. Kollock of Charleston, urging that a like action be taken by his own State.

That there should be such a legislation no one will deny when he reads the statistics of the causes of blindness in our different asylums. Many a bright child has been doomed to a life in such an institution through the delay and negligence of not having had the eyes properly attended to when suffering in its infancy from purulent ophthalmia. Credé's method has done a wonderful amount of good, but there yet remains more to be done. At the last meeting of our State Medical Association this matter of legislation was brought before that body and was unanimously referred to the proper committee, with instructions for a careful consideration of the same. We trust that this committee will make a favorable report at the meeting next April, that active steps will be at once taken. Such legislation would remove from our State some of its unnecessary expenses and, at the same time, keep from the view of the public many of those unfortunates who seek alms on our street corners.

